# OECD workshop consensus report: Ethical considerations with respect to human derived products, specifically human serum, in OECD test guidelines

**DOI:** 10.3389/ftox.2023.1140698

**Published:** 2023-02-27

**Authors:** Miriam N. Jacobs, Jan M. Bult, Kevin Cavanagh, Christophe Chesne, Nathalie Delrue, Jianan Fu, Emma Grange, Ingrid Langezaal, Dominika Misztela, Jenny Murray, Martin Paparella, Gilly Stoddart, Torsten Tonn, Carol Treasure, Masaaki Tsukano, Rosemary Versteegen

**Affiliations:** ^1^ UK Health Security Agency, Radiation, Cheimcals and Environmental Hazards, Chilton, OXON, United Kingdom; ^2^ PPTA, Brussels, Belgium; ^3^ Non-Clinical Issue, UK National Health Service—Blood and Transplant, Bristol, United Kingdom; ^4^ BIOPREDIC International, St Gregoire, France; ^5^ OECD Secretariat, Paris, France; ^6^ PAN-Biotech GmbH, Aidenbach, Germany; ^7^ Cruelty Free International, London, United Kingdom; ^8^ European Commission Joint Research Centre, Ispra, Italy; ^9^ Life Science Group Ltd, Sandy, United Kingdom; ^10^ Institute of Medical Biochemistry, Medical University of Innsbruck, Innsbruck, Austria; ^11^ PETA Science Consortium International e V, Stuttgart, Germany; ^12^ Med Fakultät Carl Gustav Carus, TU Dresden, Dresden, Germany; ^13^ XCellR8 Ltd, Cheshire, United Kingdom; ^14^ Ministry of Health, Labour and Welfare, Tokyo, Japan; ^15^ International Serum Industry Association, McHenry, MD, United States

**Keywords:** human serum, *in vitro* test methods, human derived reagents, animal-free, xenofree, ethics, human platelet lysate, human serum albumin

## Abstract

The ethical needs and concerns with use and sourcing of human materials, particularly serum, in OECD *in vitro* test guidelines were explored in a dedicated international workshop held in 2019. The health-related aspects of the donation procedure, including tissue screening, donor health, laboratory work health protection, permission from the donor for commercial use, payment of the donors and the potential for exploitation of low-income populations and data protection of the donors; supply, availability, and competition with clinical needs; traceability of the serum and auditability/GLP needs for the Test Guideline Programme, were examined. Here we provide the recommendations of the workshop with respect to the use of human serum, and potentially other human reagents, specifically with regard to test method development for OECD Test Guideline utility as part of the Mutual Acceptance of Data requirement across all OECD member countries. These include informed donor consent terminology, a checklist of human serum information requirements to be included with the Good Laboratory Practise report, and suitable sources for human serum to ensure waste supplies are used, that can no longer be used for medical purposes, ensuring no competition of supply for essential medical use.

## 1 Introduction

*In vitro* test methods used for chemical hazard assessment, can contribute relevant mechanistic data for assessing effects on human health, wildlife or the environment, both within constructs such as the Adverse Outcome Pathway (AOP), and as individual and combined test methods within OECD Test Guidelines (TGs), that are part of the Mutual Acceptance of Data principle. As the shift from *in vivo* animal testing to ‘New Approach Methods’ or ‘Non-Animal Methods’ (NAMs) increases momentum internationally, the drive to wholly replace animals in chemical hazard assessment has warranted closer inspection of the animal derived reagents used in *in vitro* testing methods.

Generally, OECD *in vitro* TGs are alternatives to *in vivo* animal testing and have greatly reduced the use of animals but cannot claim to be wholly “animal”- or “xeno—free” as they frequently include animal derived products. Reviewing the current TGs for human health, highlighted the fact that in addition to blood serum, a range of products of human origin are used, including tissues, primary cells, enzymes, collagen and antibodies. For test methods intended to be proposed to the TG Programme, human microsomes are used and other methods in development use human S9 liver extract—addressing the need to incorporate human-relevant metabolism into *in vitro* methods as highlighted by the OECD (Jacobs et al., 2008; OECD, 2008; Jacobs et al., 2013) and EURL ECVAM (Coecke et al., 2006; Bartnicka et al., 2021). Fetal bovine serum (FBS) is the most commonly utilised supplement to cell culture medium, typically used at concentrations between 5% and 10%.

To develop wholly human based test methods that are more human relevant and to avoid the use of FBS and other animal sourced material, some *in vitro* test methods are being explored or adapted for TG use without animal derived reagents. In 2017, the United Kingdom submitted a proposal to the OECD TG Programme for the adaptation of several *in vitro* test methods on skin sensitisation, to animal/xeno-free conditions (Belot et al., 2017). This proposal included the use of human serum as an alternative to the traditional cell culture media that includes FBS, and following supporting validation work, was successfully included in TG 442D by the OECD in 2018, and later updated in 2022 (OECD, 2022).

However, the project proposal and TG adaptation triggered a thoughtful debate within the Working Group of National Coordinators to the OECD Test Guideline Programme (WNT) in relation to the ethical use of human-derived products and reagents in *in vitro* TGs. It was recognised by the WNT and confirmed by the Joint Meeting in 2018 that the ethical sourcing of human-derived material (e.g., serum, blood, human liver microsomes, *etc.*) in OECD TGs would merit a broader discussion and that a dedicated workshop with subject matter experts would be useful in this respect, particularly as globally there are no existing regulations concerning blood products for the production of human serum, for experimental and commercial use. Box 1

BOX 1 Terminology definitions box: **Blood:** Whole blood collected from a human donor and processed either for transfusion or for manufacturing uses.

**Blood Components:** Therapeutic constituents of human blood (red cells, white cells, platelets and plasma) that can be prepared by various methods.

**Human Serum:** The fluid portion of the blood obtained after removing of fibrinogen and other clotting factors. It is used for many important applications, including human cell culture, drug testing, tissue typing, and cell therapy research. Human blood serum does not contain white blood cells, red blood cells, or platelets.

**Human Plasma:** The fluid portion of blood that can be separated from whole blood or collected through plasmapheresis and which contains clotting factors. It is intended either for transfusion purposes or for the manufacture of plasma derived medicinal products (PDMPs) or other manufacturing uses, such as *in vitro* medical devices.

**Recovered Plasma:** Plasma recovered from a whole blood donation and used for manufacturing uses.

**Source Plasma:** Plasma obtained by plasmapheresis intended for further manufacturing uses.

**Plasmapheresis:** A process where human plasma is obtained either for transfusion or for manufacturing purposes and cellular blood components are returned to the donor during or at the end of the donation process.

Sources: WHO: https://www.who.int/news-room/fact-sheets/detail/blood-safety-and-availability; https://www.who.int/publications/i/item/9789240021815

Accessed 29 September 2022

Having conducted a targeted review, an issues paper was developed and published in the scientific literature (Jacobs et al., 2019) in advance of the workshop. The intention of this paper was to frame the discussions and structure how to develop the solutions needed, with respect to the commercial use of human serum in TG protocols. The discussion on the ethical issues concerning the sourcing of FBS is discussed elsewhere (OECD, 2018; van der Valk et al., 2018; Versteegen et al., 2021; Weber et al., 2021), and was not the subject matter of the workshop nor this report. Our focus herein is with respect to the ethical issues related to the supply of human blood products for cell culture.

The core framing elements with specified actions identified in Jacobs et al., 2019, included direct approaches to national blood collection centres for the further examination of consent forms to understand current “Permission from Donor” and data protection communications.

A critical practical concern with respect to assuring sufficient supply of reagents of human origin from more than one commercial source, so that there is both no “monopoly” in supply, but also so that supply can be ensured, needs to ensure that the clinical blood and serum supply chain is protected such that alternative uses are not competing with patient medical needs. Here the specific action was to work out how to ensure that wherever possible, blood donations that would otherwise be considered waste and disposed of, should be the priority source for the production of human blood products, assuming that they meet all the requirements as a raw material. Furthermore, that there should be systems in place to ensure that there is no competition with clinical and therapeutic applications.

Here we describe and report on the solutions and recommendations addressing the objectives of the workshop, by a taskforce of the OECD member country nominated experts and invited experts. The workshop participants included government representatives, national blood collection agencies and experts, contract research organisations, developers of cell culture reagents and defined media, animal welfare non-governmental organisations (NGOs) representing the International Council on Animal Protection in OECD Programmes (ICAPO), serum product traceability experts, international serum industry association experts as well as the plasma collectors and fractionators’ industry representatives.

The workshop principally focused on the use of human blood products and how to make sure it is used under ethical conditions in *in vitro* TGs. However, some of the recommendations are also applicable to other human reagents. During the workshop, a more comprehensive understanding regarding the organisation and development for the traceability of FBS and human blood product supply was gained. Upon investigation as to how to avoid competition of use with the medical sector, recommendations were developed and elaborated.

## 2 Development of animal component free cell culture media

Cell culture is a widely used technique for both pharmaceutical and life science research and industry, and for the development of *in vitro* TGs that can address key human health and environmental endpoints, especially with regard to mechanistic understanding. The selection of an optimum growth medium for cell culture is a critical step in this process. Growth medium, or cell culture medium, is a complex formulation of organic and inorganic salts, amino acids, vitamins, hormones, attachment factors and growth factors. Typically, hormones, attachment- and growth factors are obtained by the addition of sourced serum. The addition of serum reduces shear-stress on the cells, and it also improves the pH buffering capacity of any medium. Replacement materials providing these benefits must also be considered.

The complexity of the formulation of cell culture media presents a number of challenges in order to fully optimise the various components. Historically, traditional cell culture media, such as RPMI 1640 and DMEM, were developed for low density cultures on a small scale. They usually require the addition of serum to obtain conditions for cell maintenance and growth. The replacement of serum in cell culture requires the addition of synthetic sources of the complex mix of amino acids, proteins, vitamins, carbohydrates, lipids, hormones, growth factors, trace elements and a myriad of other compounds naturally occurring in serum. Research into animal protein-free media dates back to the 1940s with the development of a chemically-defined media by Philip R. White with the first serum-free media being developed in the late 1970s (Yao and Asayama, 2017).

In the 1980s researchers started investigating the use of recombinant growth hormones produced in non-animal vectors, but it was not possible to produce proteins using these techniques. Instead, animal cells were used to produce recombinant proteins.

Since this time, efforts have been concentrated on conducting research to optimise the composition of cell specific media by monitoring the changes in concentration of components, genomic and proteomic-based approaches, and modifications to the producing cell lines themselves. A lot of commercial culture media are developed to optimize the growth rate, cell density or cellular productivity of *in vitro* cell culture, whilst the intention of cell culture for *in vitro* test method development, is to provide a conducive physiological basis simulating or reconstructing *in vivo* conditions. There are increasingly concerns about the basic biochemical properties of commonly used culture media, which might not be suitable for *in vitro* modelling (McKee and Komarova, 2017).

On the other hand, physiologic media, which provides better modelled *in vivo* conditions, has been proposed to be more suitable for *in vitro* studies (Cantor, 2019).

Serum-free media are difficult to formulate and whilst there have been successes for specific cell lines, there is not a universal replacement product. The development of serum-free culture media involves the testing of a variety of different combinations of essential ingredients and serum substitutes, and this requires the optimisation of a culture medium for every cell type at each stage of the cell development or manufacturing process. For all these reasons, the composition of the various culture media adopted for manufacturing purposes by the biopharmaceutical industry are not always commercially available and are (often) maintained as trade secrets.

In the issues paper (Jacobs et al., 2019), it was noted that alternatives to serum, particularly chemically defined media, are also not straightforward or quickly achievable. Indeed, the GIVIMP document (OECD, 2018) acknowledges that: for many cell types, serum-free or chemically defined media do not yet exist, and their development can be time-consuming and expensive. The document describes it though as a longer-term ideal:’ Furthermore, it is recommended to develop new *in vitro* methods with a serum-free, chemically-defined medium, to avoid potential sources of uncertainty that may be introduced by using animal serum.”

The current requirements for manufacturing in the biopharmaceutical industry require media that will sustain cells in high-densities and deliver increased production yields. Typically, these media are required to be serum-free or animal component-free and require a much higher concentration of nutrients leading to challenges in the optimisation of these products. The manufacturing processes themselves can affect both the choice of media to be used and the specific approach to the optimisation of media for a specific manufacturing process.

Recently, some funding agencies and industry are now supporting the development and public availability of the formulations and ingredients of chemically defined media, for example, with a collaborative research project in the NC3Rs Crack-It Challenge 36 (co-funded by Unilever plc) aiming to test the feasibility of adapting two OECD guideline test methods to animal-free *in vitro* conditions (https://nc3rs.org.uk/crackit/animal-free-vitro https://nc3rs.org.uk/crackit/sites/innovation/files/2021-06/Challenge%2036%20_Animal%20free%20in%20vitro_Final.pdf. Accessed 25 September 2022).

### 2.1 Considerations for the use of human serum in TGs

In the face of the extensive time and expense required in the development of (patented) defined media, the use of human serum was perhaps rather naively seen as an initial quick fix solution, pending further investigation. The GIVIMP document (OECD, 2018) states that “…The use of human serum in cell culture may be expected to provide a superior *in vitro* model of human physiology compared with animal sera … ” e.g., “FCS compared to autologous (human) serum has been found to induce a more differentiated and less stable transcriptional profile in human bone marrow mesenchymal stem cells, particularly at late passages, as shown by analysis of genome-wide microarray analysis (Shahdadfar et al., 2005).”

The workshop focused mainly on the supply of human blood, considering that the health status of the donors, their protection, as well as occupational safety are in general well controlled by national regulations in OECD Member countries. In addition, issues concerning the assessment of variability of the (pooled) reagents/s test method results, is an aspect that falls within the remit of the independent validation body, or group conducting the validation of the test method and was considered to be outside the remit of this workshop and is not discussed further here.

Although the type of donation i.e.,: Compensated or remunerated *versus* so called “altruistic” donation, also referred to as voluntary non-remunerated donation (VNRD) or voluntary unpaid donation (VUD) is debated, it was not the objective of the workshop to take a position on this issue. Of course, it is important is to ensure that donors are healthy, and blood and plasma collection is safe. Chemical residues (traces from dietary environmental chemical exposure, or due to chemical incidents/occupational exposure/poisonings) in human blood sources for the production of human serum, and potential consequent experimental confounding issues were also not discussed but are acknowledged.

The workshop did not attempt to develop recommendations in relation to the protection of donors since it appears that the donation sector is well-regulated and strict controls and procedures at the blood and plasma collection level are in place in the majority of OECD Member countries. Occupational safety was not a major focus of the workshop since biosafety standards are imposed on laboratories that conduct these assays.

#### 2.1.1 Towards ethical practices for serum supply

The opinion of the workshop participants was that the following conditions should be met in order to guarantee an ethical use of human serum in *in vitro* TGs.

• Blood should come from surplus or expired blood products that could not be used in the medical sector and,
• The donor should be informed and provide consent that surplus or expired blood may be used for non-clinical scientific (including commercial) applications.

The workshop considered the conjunction of two approaches to ensure blood products does not compete with medical uses.

• Development of a traceability audit scheme for human blood products and serum,
• Identification of expired blood sources.

Different blood components (blood, serum, platelets, plasma, others) systems in place in various countries were considered. The workshop highlighted a lack of standardisation among countries regarding the various aspects of blood components supply, with two fundamentally different ways for approaching blood components supply, specifically related to the collection of human plasma, i.e., the private (e.g., in the United States) or the public sector (e.g., in France and the United Kingdom), with some countries relying on a mixed system (public-private co-existence, e.g., Austria, Czech Republic, Germany, Hungary). Private supply comes mainly from the United States with only a limited number of European countries allowing the private sector to operate in this field.

Globally, the majority (80%) collection of human plasma for manufacturing [“Source Plasma,” to be fractionated into plasma-derived medicinal products (PDMPs)] or to be used in other applications, such as in in vitro diagnostic devices is carried out by private companies. Some of these companies are members of the Plasma Protein Therapeutic Association (PPTA) (https://www.pptaglobal.org/), an industry organisation representing private sector collectors of plasma and producers of plasma-based and recombinant biologic therapeutics which develops voluntary standards for collection, testing, manufacturing and others common to their members. In the European region, plasma collection, with the exception of four European Union countries (https://www.euneedsmoreplasma.com/images/plasma-donation/chap2.pdf, Accessed 18 October 2022) and the Ukraine, is organised by public blood establishments, some of which provide “Recovered Plasma” (plasma separated from whole blood) for manufacturing use to the private industry.

For the production of human serum, blood and plasma donations are generally pooledto increase the homogeneity and representativeness—Serum batches can contain hundreds of different individual donations, each one of which needs to be documented in an anonymous (GDPR) documented fashion. This may overcome specific issues in relation to the potential for factors contained in the blood that might affect the results of the tests. Such factors include but are not limited to; medicines taken by donors, infection history, diet, age, gender, *etc.*

The traceability of human blood outside the medical sector, that is for research and TG related activities, was not considered very efficient and it is currently problematic to know whether human serum bought from a private supplier comes from expired blood or from blood that could have been used for medical use, thus potentially creating a situation of competition between medical use and other uses. To improve this situation, a robust traceability system was recommended that would provide information on the type of supply and direct the choice of a testing laboratory when looking for a supplier. By taking this approach, the responsibility would be placed upon the testing laboratory to incentivise its serum supplier to provide blood, that is outside or expired from therapeutic use (Table 1). It was considered critical that responsibilities are shared between the serum supplier and the end user testing laboratory.

**TABLE 1:** Key considerations for information requirements concerning the origins of human serum and human reagents used in *in vitro* cell culture for OECD Test Guideline purposes to be submitted with the test results, to regulatory authorities.

Parties responsible for derivation and use of human blood derived products	Ethics and safety issues: Documentation needs	All materials of human origin	Additional comments/observations
Nationally licensed/authorised blood collection centre or equivalent and geographical location	Source: surplus/expired	+	
	Sex	+	
	Payment or altruistic donation from nationally inspected blood collection centers and equivalent, if authorised/licensed	+	
	Number of donors	+	
	Health status	+	
	Quarantine results	+	
	Specific pre-treatment	+	
	Biosafety classification	+	
	Organ/tissue of origin	+	
	Isolation technique	+	
	Date of isolation/extraction	+	
	Operator	+	
Supplier	Informed consent paperwork holding	+	
	Material transfer agreement	+	
	QC testing	+	
	Shipping conditions	+	
	Certificate of issue	+	
	Import authorisation, if relevant (sometimes not possible)	+	
	State of material on arrival	+	
	Biosafety classification	+	
	Certificate analysis	+	
	Demonstrated membership of traceability audit scheme, when available	+	
Test method/TG user	Evidence of and compliance with all of the above for completion of documentation required for submission of test results to Regulatory body	+	
GLP monitoring regulatory authority	Compliance check that ethical checks have been carried out in accordance with recommendations and GLP principles	+	
Data submitter	Similar to a GLP certificate, a statement that the study conducted complies with all of the above for completion of documentation required for submission of test results to Regulatory body	+	
Study receiving regulatory authority	Similar to GLP certificate: a statement that the study conduct complies with all of requirements listed above		

+ = required if of human origin.

From a testing laboratory perspective, some concern was expressed that suppliers may not be able to provide this level of information, if they do not run traceability programmes within their businesses. There was also testing laboratory concern that inclusion of a traceability scheme could increase the cost of the human serum, and thus the cost to customers.

**FIGURE 1:**
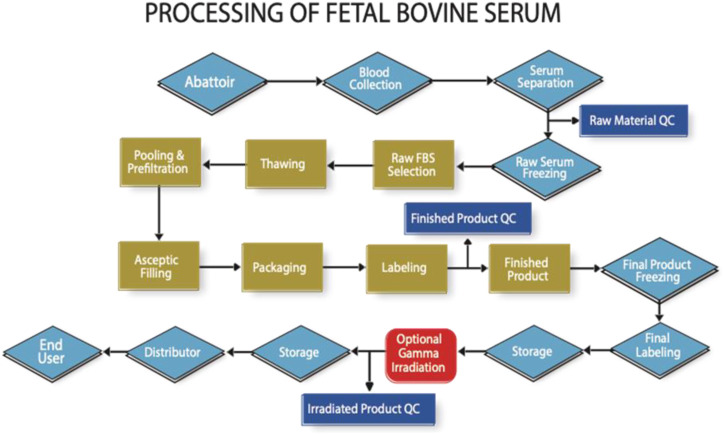
High level flow chart indicating the key processing and auditable steps in the manufacture of FBS (Note that in practise there are many more steps involved to reach each milestone indicated in this figure).

However, traceability auditing does already exist for FBS as shown in Figure 1. It is feasible to do the same for human material, and thus not compromise ethical and safety considerations for economic reasons.

#### 2.1.2 Traceability of biological samples

**FIGURE 2:**
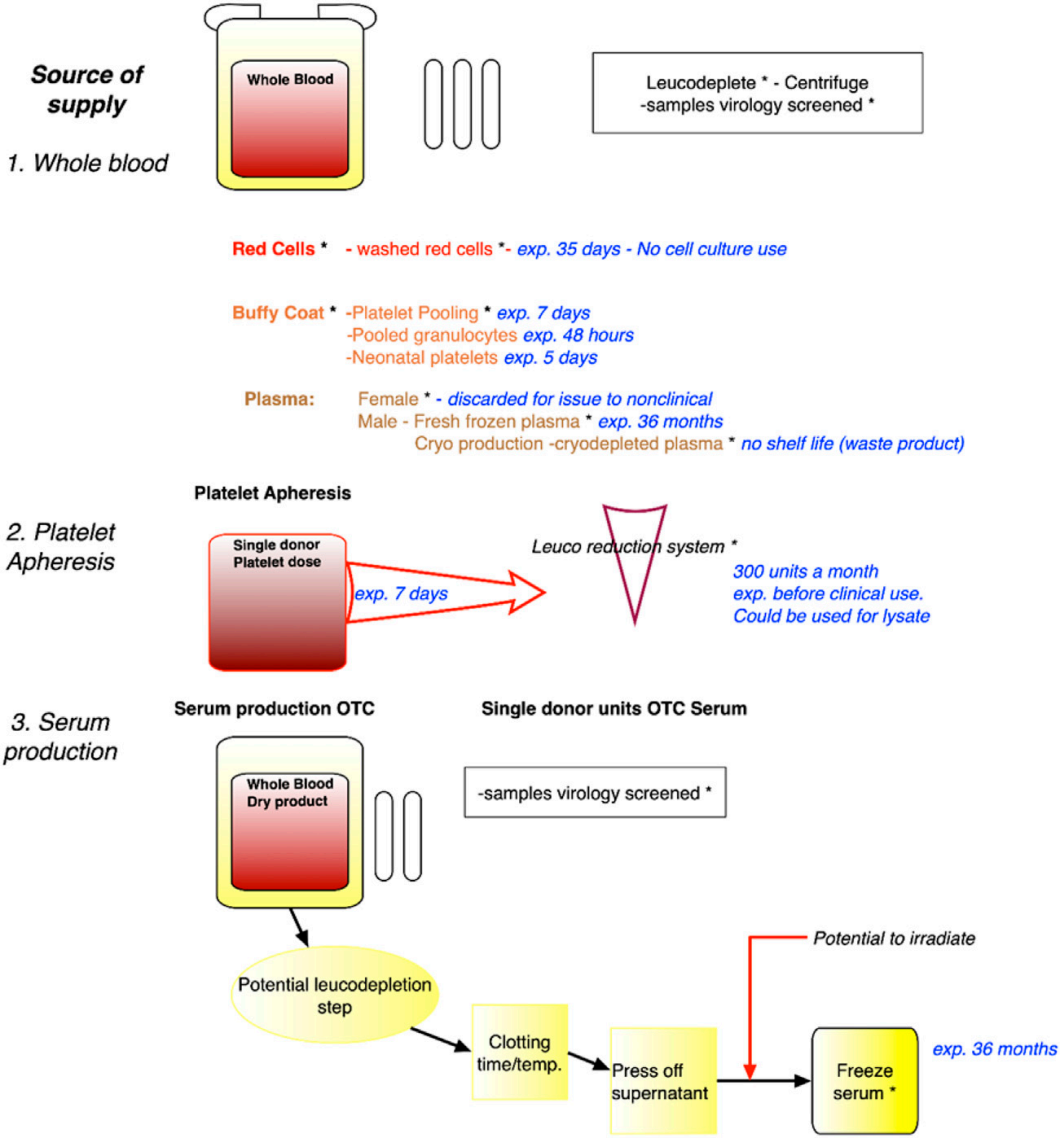
Identification of sources of expired blood no longer of use to the medical/clinical sciences. Legend. Sources of blood, including whole blood, blood fractions, platelet apheresis and serum production, with expiry dates such that there will not be any competition with medical use. Safety virology and irradiation steps are indicated. Whilst red blood cells or plasma will not be used for cell culture, platelet lysates and serum can be used. exp. = expired; OTC = Off The Clot.

Several sources of blood that would not compete with the medical use were identified at the workshop as developed in Figure 2.

These sources can be collected from blood centres, e.g., plasma that does not qualify for medical use, blood collected from volunteers excluded from the therapeutic domain or expired products. Regarding the latter, the example of platelets concentrates that have limited shelf life (i.e., 5 days) was of particular interest. Platelets concentrates that otherwise would be discarded may serve as alternative sources as cell culture substitute. Blood can be obtained from medical leftovers such as blood drawn from hemochromatosis patients (although some countries now allow this blood to be used in the medical sector). Indeed, until recently, the French example of the need for some populations with genetic blood disorders, specifically haemochromatosis, where there is a build-up of iron overload in the blood, and blood (removal) drawing is a necessary therapeutic route, has been a relevant source of human serum that does not enter the medical sector. Patients appreciated that their blood was of scientific value, and not wasted in the disposal process. However, the French Blood Establishment (EFS), the public, non-profit monopoly for blood acquisition and processing, has begun to use this source of blood for direct medical use, or for its processing at LFB, a pharmaceutical company specialising in biological medicinal products including blood derived therapeutics. It was further noted that in France, biological reagents for industrial research and development can also be obtained from 1) Clinical laboratories (collection of extra tubes and tube remnants is authorised for Non-Interventional Research), and 2) Specialised clinics collecting bloods from volunteers identified as in a clinical trial.

The collection of these types of products, in conjunction with the development of a traceability audit scheme, as developed by the ISIA in conjunction with Oritain Global Ltd. for the ISIA, on the basis of previous traceability work for food products were seen as an efficient and most ideal path forward to assist in ethical source selection, that would not compete with the medical sector.

Whilst not a financial audit, the process addressed in Figure 1, allows for confirmation of the cost at the beginning and end of each step. Audits are performed by specially trained auditors to a detailed audit protocol, where the entire focus is the confirmation of the identity of the material from abattoir to end-user. The development of an analytical method using trace element analysis to determine the country of blood collection has proven to be a helpful adjunct to the audit process (https://www.serumindustry.org/uploads/cms/nav-42-5cdb195be2c5b.pdf, Accessed 18 October 2022). Pilot experiments have shown that this methodology has the potential to be adapted to human serum, where it could be used to determine where in the world the serum was originally collected.

That some suppliers are unable to provide this level of detail about the human serum material is because they do not run traceability programmes within their business and are therefore unable to provide the level of detail requested. Without that level of control purchasers need to consider the suppliers that they would want to use—it is very much a case of “buyer beware.” Sourcing from a large, multi-national life science supplier, for instance, is not a guarantee that one can be sure about where the product is being sourced as it may have passed through a number of manufacturing entities before reaching the end user. The workshop participants strongly recommend that human material is sourced from companies with a commitment to having traceability certification and systems in place, so that the level of detail recommended here can be provided to the customer. Without this careful attention, the inclusion of human serum and materials in OECD TGs will continue to be problematic and potentially not acceptable under the Mutual Acceptance of Data agreement, for some OECD member countries.

#### 2.1.3 Screening for viral contamination

All blood and plasma donors are health screened, using extensive safety check questionnaires, and additional blood samples are taken for safety screening. Any blood donation that reacts to these tests is not used. All commercial human serum, HSA and hPL products are screened for viruses including HBsAG (Hepatitis B Surface Antigen), HIV-1/2 (Human Immunodeficiency Virus Type 1 and 2) HCV (Hepatitis C Virus) and syphilis using internationally approved methods. Final pools of plasma used for manufacturing into PDMPs are also tested for HCV RNA, HBV (Hepatitis B Virus) DNA, HTLV-1/2 (Human T-Lymphotropic Virus) and HIV-1/-2 RNA and in some instances also Parvovirus B19.

They are also screened for *mycoplasma* infection. However, the question remains, that unidentified viruses are not detected. Paraphrasing Donald Rumsfeld, “We do not know what we do not know” and we do not have suitable tools to identify unknown viruses. Only when they become a health issue, and they are characterised, are we able to develop the diagnostic and screening tools. The SARS-1 and COVID-19, ZIKA and monkey pox viruses, for example, would not have been routinely identified at the time of the workshop and still now, with current screening processes for human reagents used in TG development. The prion related Bovine Spongiform Encephalopathy (BSE) crisis in the 1980s led to the exclusion of United Kingdom blood donors from European and other international blood banks for many years.

It should also be noted that the use of bovine serum provides a level of viral biosecurity as very few bovine viruses can grow or infect human cells. The exception would be bovine leukaemia virus. The application of Gamma Irradiation to FBS as a viral load reduction method has been extensively studied and documented (https://www.serumindustry.org/gamma-irradiation, accessed 12 October 2022). Gamma irradiation is also a suitable risk mitigation step in the use of human serum and should be considered when using human derived materials. Heat inactivation is not considered a risk mitigation step for adventitious agents and is usually carried out to denature other compounds within serum.

Ideally all cell culture work with animal and human cells should be conducted at least within biosafety hazard level 2 conditions, which is considered protective for moderate hazard levels including, for example, Hepatitis B virus, human immunodeficiency virus (HIV), *Salmonella*, and Toxoplasma (https://www.cdc.gov/labs/pdf/SF__19_308133-A_BMBL6_00-BOOK-WEB-final-3.pdf, accessed 7 November 2022).

#### 2.1.4 Permission from the donor for commercial use and related considerations

There are major global differences in processes for blood donation. In the United States, donors are financially compensated, and provide signed consent forms. In Europe, donors are generally not financially compensated. However, this is debatable, as a fixed-rate allowance is permitted in four European Union countries (Austria, Czech Republic, Hungary and Germany), and consent forms are provided to and signed by the donor. With respect to the UK Human Tissues Act 2004: factors considered relevant when considering whether consent is legally required for the removal and use of human material, only scheduled purposes defined in the Act are within scope. Where material such as human serum is donated by the living for performance assessment or quality assurance purposes, the consent requirements do NOT apply, as these are not considered to be scheduled activities under the Act. Human serum is normally not considered as “relevant material” as centrifugation removes intact human cells from the resulting medium. In Japan, there is a shortage of blood donations. Blood for production as a component of blood derived products, are partly purchased from overseas.

Good practise is needed to ensure that mechanisms are in place in the source country for obtaining consent, so that the human blood products can be used for the generation of data with OECD *in vitro* TGs. The generation of TG data becomes a commercial research activity when a company performs a study for a client. Following direct approaches to collection centres and further examination of consent forms, the workshop participants agreed that the model of the United Kingdom National Health Service (NHS) Blood and Transplant (NHSBT) and the German Red Cross text information, specifically to indicate that the donation may not be used for clinical use only was sufficient. The workshop supported that a clear definition would be used for non-clinical use, i.e., left over samples, expired blood, and that the term “Industry and academic research” should be used to describe non-clinical use. The NHSBT describe how blood may be provided for non-clinical use (https://www.blood.co.uk/why-give-blood/how-blood-is-used/non-clinical-use/ accessed 18 October 2022).

An example of suitable and current text based on that provided to the workshop by the United Kingdom NHSBT and German Red Cross is:

“In some cases, we are unable to use your donation for direct transfusion to patients. This may be for a number of reasons including test results, processing issues or information we receive after donation.

As part of our commitment to a high-quality service, we sometimes use donations for laboratory work, education, training, research and development, which may include DNA studies and export. Donations may also be used in the preparation of healthcare related medicinal products, within NHSBT or by other organisations which could be outside the United Kingdom. These are essential for effective patient care. If we use your donation for any of these purposes, we will ensure that:

• ethical approval is obtained where appropriate:
• there are no implications for your health or welfare.
• you cannot be identified; this includes any work involving DNA studies.
• any income generated is used for the benefit of NHSBT and the wider NHS. NHSBT is a non-profit organisation.
• no DNA analysis is performed that may identify you without your specific/explicit consent.

To stay in touch, we may compare details with central NHS records or those of other data processors. We are committed to protecting your confidentiality and to meeting our responsibilities under the Data Protection Act 2018, which constitutes the United Kingdom implementation of the General Data Protection Regulation (GDPR).” (Sources: https://nhsbtdbe.blob.core.windows.net/umbraco-assets-corp/23778/2122-0067-donor-consent-information-leaflet-blood-final.pdf and https://nhsbtdbe.blob.core.windows.net/umbraco-assets-corp/23779/2122-0068-donor-consent-information-leaflet-platelets-and-plasma-final.pdf accessed 18 October 2022).

The information provided to donors also explains that using expired blood for non-medical purposes reduces disposal and waste expenses for the National Health Service.

As is now normal practise, the donor consent form and further pooling and data processing includes privacy/GPDR considerations, and this needs to be protected with regard to traceability.

## 3 Summary of the outcome of the workshop: A 2-step recommendation

Recognising that it will take time before a traceability scheme can be developed for human serum, the workshop developed a two-step recommendation, ultimately intended to address a more optimum longer-term recommendation, as well as elements of a short-term recommendation, that can be implemented during the transition.

The short-term recommendation was the implementation of three deliverables developed during and finalised shortly after the workshop. This temporary solution is proposed to provide guidance to CROs during the transition. The three deliverables are the following:

• Checklist of information requirements to be documented by the testing laboratory (Table 1): The objective of this checklist is to ensure that the human blood products have been sourced in an ethical manner. It consists of a list of information requirements that would need to be documented together with the test results, for inspection by the respective responsible (GLP) authorities. Required information concerns the origins of human serum and human reagents and are derived and expanded upon from the OECD Guidance Document on Good *In Vitro* Method Practice (GIVIMP) (OECD, 2018) and initial issues paper (Jacobs et al., 2019) Table 1.
• Donor informed consent: Based on the example of NHSBT, the workshop considered the development of a harmonised proposal for donor informed consent, in order to transparently inform the donor that a donation may not be used for clinical use only. There was support that such a text should be further refined in order to define what would be used for non-clinical use, i.e.,: Left over samples, expired blood, and should also describe what non-clinical uses are covered.
• Identification of sources of expired blood: The workshop participants developed a flow diagram that describe the various characteristics of each blood fraction, including related production processes, shelf life etc., to identify and enable the time points at which blood that can no longer be used for medical therapeutic purposes, can be regarded as an appropriate source for non-clinical applications. This systematic identification can be the basis for subsequent recommendations regarding potential sources of serum for non-clinical use and is shown in Figure 2.

The longer-term recommendation is to encourage the continued development of a traceability audit scheme. It is proposed that this traceability audit scheme is based on the well-established model developed by the International Serum Industry Association (ISIA) for FBS. ISIA has developed voluntary standards and provides certification to companies that meet these standards. It was noted that most human serum producers from the private sector (mainly represented by the Plasma Protein Therapeutic Association or PPTA) are also members of the ISIA, which may facilitate the process.

## 4 Conclusion: The way forward for the ethical use of human serum in OECD TGs, and the development of alternatives

Following the outcome of the workshop described above, in April 2019, the WNT supported further work towards the implementation of a traceability audit scheme in order to guarantee a stable supply of human serum that would not compete with the medical use. The WNT also supported the short-term recommendations of the workshop, i.e.,: Development of a checklist to make sure the serum used has been sourced in an ethical manner, development of a donor informed consent model, and identification of expired blood sources as potential sources of serum for *in vitro* assays.

Japan noted, however, in the proposed supply chain, that is outside the medical sector, the safety of all the persons involved, from donors, staff in sample collection facilities/donation facilities to the users of the test method is paramount. Accidental exposure to human serum could have more serious health consequences than exposure to FBS which is currently more strictly controlled, and which seems less likely to contain human-specific communicable pathogens; therefore, it is necessary to take essential measures including discrimination by labelling, separate waste disposal of contaminated materials, training of end users, *etc.*

Therefore, in the future, the recommendations proposed should overcome some of the ethical concerns associated with the use of human derived products in OECD TGs, and it is noted however that numerous challenges remain which need to be taken into consideration and addressed very carefully. The need for chemically defined media as an option to replace animal/human serum in *in vitro* test methods is apparent, and these technologies need to be progressed and considered in the future.

The post workshop finalisations of the recommendations were conducted through 2019 and early 2020, and include

1. A detailed checklist (Table 1) that following WNT consultation, can be proposed as a basis for inclusion with the submission of the test report, for the inspection of GLP (and regulatory) authorities, where human serum is used;
2. Encouraging the use of the donor informed consent model by blood collection centres OECD-wide;
3. A final scheme for recommendations regarding potential sources of expired/surplus blood that would not compete with the medical use (Figure 1);
4. Working towards the development of a traceability scheme for human serum supply

In early January 2020 the global COVID pandemic led to a hiatus in the continuation and publication of this work, not least because the evident hazards of unknown viruses were evident for all to see and experience.

However human serum use for *in vitro* TGs remains an important area of test method development by many stakeholders, and *in vitro* (pre)validation projects are currently being undertaken where human serum, microsomes and primary cells are being incorporated. There is an evident need still for ethical protections to be in place, with respect to these human sourced *in vitro* method components, and this workshop has provided solutions as to how this can be addressed. Next steps will include looking at how to put these solutions into practice to continue to facilitate the inclusion of human-derived reagents in *in vitro* OECD TGs.

More recently established methods are already based on fully defined media, like the RTgill-W1 cell-line based OECD TG249 and also the hiPSC based models for the *in vitro* battery for developmental neurotoxicity testing (characterized within the respective draft OECD Guidance Document). Other work towards individually crafted and financially demanding chemically defined media is currently in the process of being developed as proof of concept for two specified *in vitro* TGs (TG487 and TG455), this will still take quite some time, despite substantial investment. As with all test method development work, the transition to chemically defined media is a question of funding and resources, the likelihood of developing chemically defined media for each and all the *in vitro* TGs that use FBS will not be accomplished in the near future, and ideally it will be the most highly used *in vitro* TGs that are the first 3Rs funding targets in this regard. The considerations presented here, concerning the ethical sourcing of serum for OECD TGs are also applicable to general research. Thus, it is important to ensure that there are processes in place for the use of human serum in cell culture, and that end-users look at how they can address this currently unmet need.

Finally, the recommendations and solutions discussed herein on the use of human serum may be suitable for discussion with policymakers and legislators, particularly in light of a potential increase in the global demand for human serum for medical, scientific and regulatory sectors.

## Data Availability

The original contributions presented in the study are included in the article, further inquiries can be directed to the corresponding author.
